# 1-(Biphenyl-4-ylcarbon­yl)-3-(4-nitro­phen­yl)thio­urea

**DOI:** 10.1107/S160053681103426X

**Published:** 2011-08-27

**Authors:** M. Sukeri M. Yusof, Sze Ting Wong, Bohari M. Yamin

**Affiliations:** a Department of Chemical Sciences, Faculty of Science and Technology, Universiti Malaysia Terengganu, 21030 Kuala Terengganu, Terengganu, Malaysia; b School of Chemical Sciences and Food Technology, Universiti Kebangsaan Malaysia, UKM 43500 Bangi Selangor, Malaysia

## Abstract

In the title compound, C_20_H_15_N_3_O_3_S, the two benzene rings of the biphenyl group form a dihedral angle of 40.11 (15)°. The conformation of the mol­ecule is *trans*–*cis* and is stabilized by two intra­molecular N—H⋯O and C—H⋯S hydrogen bonds. In the crystal structure, the mol­ecules are linked by weak π–π stacking inter­actions [centroid–centroid distance = 3.991 (2) Å].

## Related literature

For related structures, see: Arif &Yamin (2007 ▶); Yamin & Arif (2008 ▶). For standard bond lengths, see: Allen *et al.* (2003 ▶).
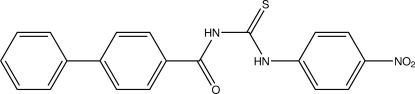


## Experimental

### 

#### Crystal data

C_20_H_15_N_3_O_3_S
*M* *_r_* = 377.41
Monoclinic, 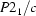

*a* = 12.154 (2) Å
*b* = 9.4509 (18) Å
*c* = 17.471 (3) Å
β = 118.133 (9)°
*V* = 1769.7 (5) Å^3^
*Z* = 4
Mo *K*α radiation
μ = 0.21 mm^−1^
*T* = 298 K
0.38 × 0.14 × 0.07 mm

#### Data collection

Bruker SMART APEX CCD area-detector diffractometer
Absorption correction: multi-scan (*SADABS*; Bruker, 2000 ▶) *T*_min_ = 0.925, *T*_max_ = 0.986
9891 measured reflections
3116 independent reflections
2268 reflections with *I* > 2/s(*I*)
*R*_int_ = 0.043

#### Refinement

*R*[*F* ^2^ > 2σ(*F* ^2^)] = 0.060
*wR*(*F* ^2^) = 0.143
*S* = 0.88
3116 reflections
244 parameters
H-atom parameters constrained
Δρ_max_ = 0.26 e Å^−3^
Δρ_min_ = −0.22 e Å^−3^

### 

Data collection: *SMART* (Bruker, 2000 ▶); cell refinement: *SAINT* (Bruker, 2000 ▶); data reduction: *SAINT*; program(s) used to solve structure: *SHELXS97* (Sheldrick, 2008 ▶); program(s) used to refine structure: *SHELXL97* (Sheldrick, 2008 ▶); molecular graphics: *OLEX2* (Dolomanov *et al.*, 2009 ▶), *SHELXTL* (Sheldrick, 2008 ▶), *PLATON* (Spek, 2009 ▶); software used to prepare material for publication: *SHELXTL*, *PARST* (Nardelli, 1995 ▶).

## Supplementary Material

Crystal structure: contains datablock(s) global, I. DOI: 10.1107/S160053681103426X/bx2367sup1.cif

Structure factors: contains datablock(s) I. DOI: 10.1107/S160053681103426X/bx2367Isup2.hkl

Supplementary material file. DOI: 10.1107/S160053681103426X/bx2367Isup3.cml

Additional supplementary materials:  crystallographic information; 3D view; checkCIF report

## Figures and Tables

**Table 1:** Hydrogen-bond geometry (Å, °)

*D*—H⋯*A*	*D*—H	H⋯*A*	*D*⋯*A*	*D*—H⋯*A*
N2—H2*A*⋯O1	0.86	1.90	2.633 (4)	142
C20—H20⋯S1	0.93	2.55	3.186 (4)	126

**Fig. 1.**
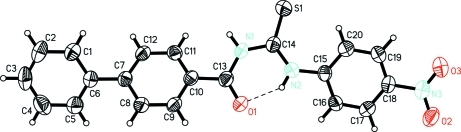
The molecular structure of (I), with displacement ellipsods drawn at the 50% probability level. The hydrogen bonds are shown by dashed lines.

**Fig. 2.**
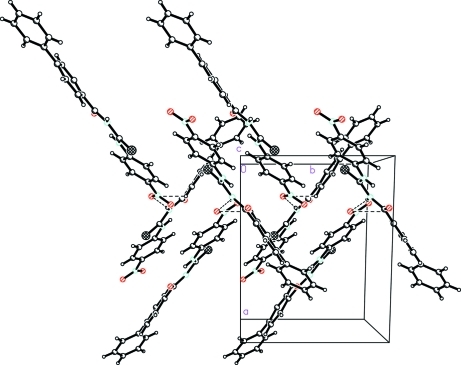
π-π stacking interactions. The centroids are linked by dashed lines.

## supplementary crystallographic information

### Comment

The title compound, (I) is analogous to the previously reported *N*-(biphenyl-4-carbonyl)-*N*'-(4-chlorophenyl)thiourea (II) (Yamin & Arif, 2008) except the chlorine atom in (II) is replaced by a nitro group. The bond lengths and angles are in normal ranges (Allen *et al.*, 2003) and comparable to those previous reported (Arif & Yamin, 2007). The benzene rings (C1—C6, C7—C12, C15—C20) and thiourea moities (C14/N1/N2/S1) are all planar with maximum deviation of 0.043 (3) Å for atom N1 from the mean plane. The dihedral angle of two benzene rings of the biphenyl group are at an angle of 40.11 (15)° smaller compared in (II)(44.23 (12)°). The central thiourea fragment makes dihedral angles with the benzene-carbonyl (C7—C12) and nitrobenzene (C15—C20) rings of 16.14 (13) and 17.75 (14)°, respectively, smaller compared in (II) (55.96 (9) and 64.09 (9)°). The conformation of the molecule is *trans*-*cis* and is stabilized by two intramolecular hydrogen bonds N—H^.^···O and C—H^.^···S interactions. In the crystal structure, the molecules are linked by weak π-π stacking interactions, Table1 & Table2, Fig2.

### Experimental

A solution of biphenylcarbomoylisothiocyanate (2.0 g, 8.4 mmol) in 20 ml acetone was added dropwise to a two-necked round-bottomed flask containing an equimolar amount of 4-nitroaniline (1.15 g, 8.4 mmol) in 20 ml of acetone. The mixture was refluxed for about 2.5 h. The light yellow solution was filtered and the filtrate allowed to evaporate at room temperature. Light yellow crystals were obtained after five days (yield 63%, m.p.: 164–166°C).

### Refinement

H atoms on the parent carbon atoms were positioned geometrically with C—H= 0.93Å (benzene) and N—H = 0.86 Å, constrained to ride on their parent atoms with *U*_iso_(H)= *xU*_eq_(parent atom) where *x*=1.2 for CH and NH groups.

### Crystal data

**Table:**

C_20_H_15_N_3_O_3_S	*F*(000) = 784
*M**_r_* = 377.41	*D*_x_ = 1.416 Mg m^−^^3^
Monoclinic, *P*2_1_/*c*	Mo *K*α radiation, λ = 0.71073 Å
Hall symbol: -P 2ybc	Cell parameters from 777 reflections
*a* = 12.154 (2) Å	θ = 1.9–25.0°
*b* = 9.4509 (18) Å	µ = 0.21 mm^−^^1^
*c* = 17.471 (3) Å	*T* = 298 K
β = 118.133 (9)°	Slab, light yellow
*V* = 1769.7 (5) Å^3^	0.38 × 0.14 × 0.07 mm
*Z* = 4	

### Data collection

**Table:**

Bruker SMART APEX CCD area-detector diffractometer	3116 independent reflections
Radiation source: fine-focus sealed tube	2268 reflections with *I* > 2/s(*I*)
graphite	*R*_int_ = 0.043
Detector resolution: 83.66 pixels mm^-1^	θ_max_ = 25.0°, θ_min_ = 1.9°
ω scan	*h* = −13→14
Absorption correction: multi-scan (*SADABS*; Bruker, 2000)	*k* = −10→11
*T*_min_ = 0.925, *T*_max_ = 0.986	*l* = −20→20
9891 measured reflections	

### Refinement

**Table:**

Refinement on *F*^2^	Primary atom site location: structure-invariant direct methods
Least-squares matrix: full	Secondary atom site location: difference Fourier map
*R*[*F*^2^ > 2σ(*F*^2^)] = 0.060	Hydrogen site location: inferred from neighbouring sites
*wR*(*F*^2^) = 0.143	H-atom parameters constrained
*S* = 0.88	*w* = 1/[σ^2^(*F*_o_^2^) + (0.0447*P*)^2^ + 3.2573*P*] where *P* = (*F*_o_^2^ + 2*F*_c_^2^)/3
3116 reflections	(Δ/σ)_max_ < 0.001
244 parameters	Δρ_max_ = 0.26 e Å^−^^3^
0 restraints	Δρ_min_ = −0.22 e Å^−^^3^

### Special details

**Table:**

Geometry. All e.s.d.'s (except the e.s.d. in the dihedral angle between two l.s. planes) are estimated using the full covariance matrix. The cell e.s.d.'s are taken into account individually in the estimation of e.s.d.'s in distances, angles and torsion angles; correlations between e.s.d.'s in cell parameters are only used when they are defined by crystal symmetry. An approximate (isotropic) treatment of cell e.s.d.'s is used for estimating e.s.d.'s involving l.s. planes.
Refinement. Refinement of *F*^2^ against ALL reflections. The weighted *R*-factor *wR* and goodness of fit *S* are based on *F*^2^, conventional *R*-factors *R* are based on *F*, with *F* set to zero for negative *F*^2^. The threshold expression of *F*^2^ > σ(*F*^2^) is used only for calculating *R*-factors(gt) *etc*. and is not relevant to the choice of reflections for refinement. *R*-factors based on *F*^2^ are statistically about twice as large as those based on *F*, and *R*- factors based on ALL data will be even larger.

### Fractional atomic coordinates and isotropic or equivalent isotropic displacement parameters (Å^2^)

**Table:**

	*x*	*y*	*z*	*U*_iso_*/*U*_eq_	
S1	0.34844 (10)	−0.26185 (12)	−0.10149 (6)	0.0687 (4)	
O1	0.3207 (2)	−0.5537 (3)	0.09461 (16)	0.0618 (7)	
O2	1.0052 (2)	−0.1773 (3)	0.30122 (17)	0.0662 (7)	
O3	0.9535 (3)	−0.0226 (3)	0.19967 (19)	0.0773 (9)	
N1	0.2547 (2)	−0.4403 (3)	−0.03407 (17)	0.0445 (7)	
H1	0.1906	−0.4296	−0.0842	0.053*	
N2	0.4557 (2)	−0.3739 (3)	0.05899 (17)	0.0473 (7)	
H2A	0.4451	−0.4353	0.0913	0.057*	
N3	0.9295 (3)	−0.1246 (3)	0.2325 (2)	0.0540 (8)	
C1	−0.3311 (3)	−0.7919 (4)	−0.1811 (2)	0.0524 (9)	
H1A	−0.3269	−0.7079	−0.2074	0.063*	
C2	−0.4442 (3)	−0.8619 (5)	−0.2122 (2)	0.0612 (10)	
H2B	−0.5157	−0.8240	−0.2578	0.073*	
C3	−0.4498 (4)	−0.9871 (5)	−0.1752 (3)	0.0674 (12)	
H3A	−0.5255	−1.0347	−0.1962	0.081*	
C4	−0.3461 (4)	−1.0434 (5)	−0.1080 (3)	0.0717 (12)	
H4	−0.3508	−1.1292	−0.0837	0.086*	
C5	−0.2324 (4)	−0.9710 (4)	−0.0758 (2)	0.0595 (10)	
H5	−0.1617	−1.0090	−0.0295	0.071*	
C6	−0.2238 (3)	−0.8443 (3)	−0.1117 (2)	0.0423 (8)	
C7	−0.1055 (3)	−0.7659 (3)	−0.0786 (2)	0.0402 (7)	
C8	−0.0235 (3)	−0.7554 (4)	0.0100 (2)	0.0468 (8)	
H8	−0.0436	−0.7995	0.0493	0.056*	
C9	0.0873 (3)	−0.6809 (4)	0.0405 (2)	0.0462 (8)	
H9	0.1410	−0.6763	0.0999	0.055*	
C10	0.1184 (3)	−0.6134 (3)	−0.0165 (2)	0.0398 (7)	
C11	0.0382 (3)	−0.6242 (3)	−0.1049 (2)	0.0435 (8)	
H11	0.0586	−0.5800	−0.1440	0.052*	
C12	−0.0709 (3)	−0.6991 (4)	−0.1351 (2)	0.0471 (8)	
H12	−0.1229	−0.7055	−0.1946	0.056*	
C13	0.2392 (3)	−0.5352 (3)	0.0202 (2)	0.0450 (8)	
C14	0.3579 (3)	−0.3590 (3)	−0.0200 (2)	0.0433 (8)	
C15	0.5734 (3)	−0.3067 (3)	0.0989 (2)	0.0407 (8)	
C16	0.6628 (3)	−0.3681 (4)	0.1744 (2)	0.0458 (8)	
H16	0.6438	−0.4499	0.1954	0.055*	
C17	0.7800 (3)	−0.3090 (4)	0.2191 (2)	0.0484 (8)	
H17	0.8405	−0.3504	0.2698	0.058*	
C18	0.8055 (3)	−0.1871 (4)	0.1869 (2)	0.0452 (8)	
C19	0.7179 (3)	−0.1240 (4)	0.1130 (2)	0.0557 (9)	
H19	0.7372	−0.0418	0.0926	0.067*	
C20	0.5997 (3)	−0.1833 (4)	0.0686 (2)	0.0561 (9)	
H20	0.5387	−0.1401	0.0188	0.067*	

### Atomic displacement parameters (Å^2^)

**Table:**

	*U*^11^	*U*^22^	*U*^33^	*U*^12^	*U*^13^	*U*^23^
S1	0.0687 (7)	0.0718 (7)	0.0517 (6)	−0.0203 (6)	0.0169 (5)	0.0114 (5)
O1	0.0406 (14)	0.0756 (18)	0.0498 (15)	−0.0105 (12)	0.0054 (12)	0.0164 (13)
O2	0.0439 (15)	0.0724 (18)	0.0605 (17)	−0.0035 (13)	0.0068 (13)	−0.0029 (14)
O3	0.0691 (19)	0.075 (2)	0.0768 (19)	−0.0285 (16)	0.0255 (16)	0.0006 (16)
N1	0.0330 (15)	0.0523 (17)	0.0412 (15)	−0.0026 (13)	0.0118 (13)	0.0020 (13)
N2	0.0439 (17)	0.0477 (16)	0.0481 (16)	−0.0050 (13)	0.0198 (14)	0.0046 (13)
N3	0.0472 (19)	0.056 (2)	0.0563 (19)	−0.0065 (15)	0.0223 (17)	−0.0103 (16)
C1	0.045 (2)	0.055 (2)	0.050 (2)	−0.0013 (17)	0.0155 (18)	−0.0062 (17)
C2	0.039 (2)	0.075 (3)	0.060 (2)	−0.0044 (19)	0.0147 (18)	−0.022 (2)
C3	0.050 (2)	0.082 (3)	0.076 (3)	−0.028 (2)	0.034 (2)	−0.032 (2)
C4	0.082 (3)	0.066 (3)	0.075 (3)	−0.026 (2)	0.043 (3)	−0.006 (2)
C5	0.055 (2)	0.058 (2)	0.058 (2)	−0.0060 (19)	0.0209 (19)	0.0014 (19)
C6	0.0398 (19)	0.0454 (19)	0.0434 (18)	−0.0018 (15)	0.0211 (16)	−0.0078 (15)
C7	0.0355 (18)	0.0381 (18)	0.0447 (18)	0.0028 (14)	0.0171 (15)	−0.0019 (14)
C8	0.0411 (19)	0.055 (2)	0.0437 (19)	−0.0005 (16)	0.0192 (16)	0.0033 (16)
C9	0.0369 (18)	0.060 (2)	0.0349 (17)	−0.0019 (16)	0.0112 (15)	0.0011 (16)
C10	0.0333 (17)	0.0415 (18)	0.0416 (18)	0.0054 (14)	0.0152 (15)	0.0004 (14)
C11	0.0423 (19)	0.049 (2)	0.0438 (19)	0.0019 (16)	0.0239 (16)	0.0039 (15)
C12	0.0409 (19)	0.056 (2)	0.0356 (17)	−0.0030 (16)	0.0112 (15)	−0.0014 (15)
C13	0.0371 (19)	0.0444 (19)	0.047 (2)	0.0019 (15)	0.0151 (17)	0.0007 (16)
C14	0.0384 (19)	0.0388 (18)	0.0474 (19)	0.0024 (15)	0.0160 (16)	−0.0037 (15)
C15	0.0370 (18)	0.0422 (18)	0.0447 (18)	−0.0019 (15)	0.0206 (16)	−0.0044 (15)
C16	0.042 (2)	0.047 (2)	0.050 (2)	−0.0038 (16)	0.0229 (17)	0.0040 (16)
C17	0.043 (2)	0.055 (2)	0.0412 (19)	0.0029 (17)	0.0151 (16)	0.0006 (16)
C18	0.0373 (18)	0.0473 (19)	0.049 (2)	−0.0056 (16)	0.0185 (16)	−0.0078 (16)
C19	0.054 (2)	0.046 (2)	0.061 (2)	−0.0100 (18)	0.022 (2)	0.0050 (18)
C20	0.046 (2)	0.054 (2)	0.057 (2)	−0.0009 (18)	0.0153 (18)	0.0103 (18)

### Geometric parameters (Å, °)

**Table:**

S1—C14	1.652 (3)	C6—C7	1.472 (4)
O1—C13	1.219 (4)	C7—C12	1.393 (4)
O2—N3	1.222 (4)	C7—C8	1.394 (4)
O3—N3	1.226 (4)	C8—C9	1.384 (4)
N1—C13	1.382 (4)	C8—H8	0.9300
N1—C14	1.391 (4)	C9—C10	1.378 (4)
N1—H1	0.8605	C9—H9	0.9300
N2—C14	1.338 (4)	C10—C11	1.387 (4)
N2—C15	1.413 (4)	C10—C13	1.492 (4)
N2—H2A	0.8601	C11—C12	1.370 (4)
N3—C18	1.457 (4)	C11—H11	0.9300
C1—C2	1.385 (5)	C12—H12	0.9300
C1—C6	1.388 (4)	C15—C20	1.379 (5)
C1—H1A	0.9300	C15—C16	1.381 (4)
C2—C3	1.365 (6)	C16—C17	1.379 (4)
C2—H2B	0.9300	C16—H16	0.9300
C3—C4	1.361 (6)	C17—C18	1.380 (5)
C3—H3A	0.9300	C17—H17	0.9300
C4—C5	1.400 (5)	C18—C19	1.363 (5)
C4—H4	0.9300	C19—C20	1.389 (5)
C5—C6	1.378 (5)	C19—H19	0.9300
C5—H5	0.9300	C20—H20	0.9300
			
C13—N1—C14	129.9 (3)	C8—C9—H9	119.8
C13—N1—H1	115.1	C9—C10—C11	118.8 (3)
C14—N1—H1	115.1	C9—C10—C13	117.9 (3)
C14—N2—C15	131.5 (3)	C11—C10—C13	123.3 (3)
C14—N2—H2A	114.3	C12—C11—C10	120.7 (3)
C15—N2—H2A	114.2	C12—C11—H11	119.6
O2—N3—O3	123.1 (3)	C10—C11—H11	119.6
O2—N3—C18	118.5 (3)	C11—C12—C7	121.5 (3)
O3—N3—C18	118.4 (3)	C11—C12—H12	119.3
C2—C1—C6	121.5 (4)	C7—C12—H12	119.3
C2—C1—H1A	119.3	O1—C13—N1	121.3 (3)
C6—C1—H1A	119.3	O1—C13—C10	121.9 (3)
C3—C2—C1	119.4 (4)	N1—C13—C10	116.8 (3)
C3—C2—H2B	120.3	N2—C14—N1	114.4 (3)
C1—C2—H2B	120.3	N2—C14—S1	127.9 (3)
C4—C3—C2	120.9 (4)	N1—C14—S1	117.7 (2)
C4—C3—H3A	119.5	C20—C15—C16	120.2 (3)
C2—C3—H3A	119.5	C20—C15—N2	123.7 (3)
C3—C4—C5	119.5 (4)	C16—C15—N2	116.1 (3)
C3—C4—H4	120.2	C17—C16—C15	120.5 (3)
C5—C4—H4	120.2	C17—C16—H16	119.7
C6—C5—C4	120.9 (4)	C15—C16—H16	119.7
C6—C5—H5	119.5	C16—C17—C18	118.5 (3)
C4—C5—H5	119.5	C16—C17—H17	120.7
C5—C6—C1	117.8 (3)	C18—C17—H17	120.7
C5—C6—C7	121.9 (3)	C19—C18—C17	121.7 (3)
C1—C6—C7	120.3 (3)	C19—C18—N3	119.0 (3)
C12—C7—C8	117.2 (3)	C17—C18—N3	119.2 (3)
C12—C7—C6	121.0 (3)	C18—C19—C20	119.6 (3)
C8—C7—C6	121.7 (3)	C18—C19—H19	120.2
C9—C8—C7	121.3 (3)	C20—C19—H19	120.2
C9—C8—H8	119.3	C15—C20—C19	119.5 (3)
C7—C8—H8	119.3	C15—C20—H20	120.3
C10—C9—C8	120.4 (3)	C19—C20—H20	120.3
C10—C9—H9	119.8		
			
C6—C1—C2—C3	−1.8 (5)	C9—C10—C13—O1	15.8 (5)
C1—C2—C3—C4	0.5 (6)	C11—C10—C13—O1	−162.5 (3)
C2—C3—C4—C5	0.7 (6)	C9—C10—C13—N1	−164.2 (3)
C3—C4—C5—C6	−0.6 (6)	C11—C10—C13—N1	17.4 (5)
C4—C5—C6—C1	−0.6 (5)	C15—N2—C14—N1	−177.0 (3)
C4—C5—C6—C7	179.7 (3)	C15—N2—C14—S1	5.4 (5)
C2—C1—C6—C5	1.8 (5)	C13—N1—C14—N2	−1.4 (5)
C2—C1—C6—C7	−178.5 (3)	C13—N1—C14—S1	176.4 (3)
C5—C6—C7—C12	139.9 (3)	C14—N2—C15—C20	16.4 (5)
C1—C6—C7—C12	−39.8 (5)	C14—N2—C15—C16	−166.6 (3)
C5—C6—C7—C8	−40.0 (5)	C20—C15—C16—C17	−1.7 (5)
C1—C6—C7—C8	140.3 (3)	N2—C15—C16—C17	−178.8 (3)
C12—C7—C8—C9	0.5 (5)	C15—C16—C17—C18	0.4 (5)
C6—C7—C8—C9	−179.7 (3)	C16—C17—C18—C19	0.5 (5)
C7—C8—C9—C10	0.7 (5)	C16—C17—C18—N3	−179.0 (3)
C8—C9—C10—C11	−1.3 (5)	O2—N3—C18—C19	176.0 (3)
C8—C9—C10—C13	−179.8 (3)	O3—N3—C18—C19	−4.9 (5)
C9—C10—C11—C12	0.7 (5)	O2—N3—C18—C17	−4.5 (5)
C13—C10—C11—C12	179.1 (3)	O3—N3—C18—C17	174.6 (3)
C10—C11—C12—C7	0.5 (5)	C17—C18—C19—C20	−0.1 (5)
C8—C7—C12—C11	−1.1 (5)	N3—C18—C19—C20	179.5 (3)
C6—C7—C12—C11	179.0 (3)	C16—C15—C20—C19	2.2 (5)
C14—N1—C13—O1	3.9 (5)	N2—C15—C20—C19	179.0 (3)
C14—N1—C13—C10	−176.1 (3)	C18—C19—C20—C15	−1.3 (6)

### Hydrogen-bond geometry (Å, °)

**Table:**

*D*—H···*A*	*D*—H	H···*A*	*D*···*A*	*D*—H···*A*
N2—H2A···O1	0.86	1.90	2.633 (4)	142
C20—H20···S1	0.93	2.55	3.186 (4)	126

### Table 2 π–π stacking interaction in (I)

Cg1 is the centroid of the C7–C12 ring, φ is the dihedral angle (°) between the planes of the rings, d is the distance (Å) between the ring centroids and Δ is the displacement (Å) of the centroid of ring 2 relative to the intersection point of the normal to the centroid of ring 1 and the least-squares plane of ring 2.

**Table:**

Ring 1	Ring 2	φ	d	Δ
Cg1	Cg1i	0.0	3.991 (2)	1.778

Symmetry code: (i) -x, -1-y, -z
